# Effect of tadalafil 5mg daily treatment on the ejaculatory times, lower urinary tract symptoms and erectile function in patients with erectile dysfunction

**DOI:** 10.1590/S1677-5538.IBJU.2016.0376

**Published:** 2017

**Authors:** Mehmet Karabakan, Ercument Keskin, Serkan Akdemir, Aliseydi Bozkurt

**Affiliations:** 1 Department of Urology, Mersin Toros State Hospital, Mersin, Turkey;; 2 Department of Urology, Mengucek Gazi Research and Training Hospital, Erzincan University, Erzincan, Turkey;; 3 Department of Urology, Faculty of Medicine, Izmir University, Izmir, Turkey

**Keywords:** Tadalafil, Ejaculation, Erectile Dysfunction, Therapeutics

## Abstract

**Objective:**

To investigate the effect of a 5mg daily tadalafil treatment on the ejaculation time, erectile function and lower urinary tract symptoms (LUTS) in patients with erectile dysfunction.

**Materials and Methods:**

A total of 60 patients diagnosed with erectile dysfunction were retrospectively evaluated using the international index of erectile function questionnaire-5 (IIEF-5), intravaginal ejaculatory latency time (IELT) and international prostate symptoms scores (IPSS). After the patients were treated with 5mg tadalafil once a day for three months, their erection, ejaculation and LUTS were assessed again. The fasting levels of blood glucose, total testosterone, low-density lipoprotein cholesterol, high-density lipoprotein cholesterol and total cholesterol were measured. The independent-samples t-test was used to compare the pre- and post-treatment scores of the patients.

**Results:**

The mean age of the 60 participants was 50.4±7.9 and the mean baseline serum total testosterone, total cholesterol, and fasting blood sugar were 444.6±178.6ng dL^-1^, 188.7±29.6mg/dL^-1^,104 (80-360) mg dL^-1^, respectively. The mean baseline scores were 2.2±1.4 min for IELT, 9.5±3.7 for IIEF-5 and 14.1±4.5 for IPSS. Following the three-month daily 5mg tadalafil treatment, the scores were found to be 3.4±1.9 min, 16.1±4.7, and 10.4±3.8 for IELT, IIEF and IPSS, respectively. When the baseline and post-treatment scores were compared, a statistically significant increase was observed in the IELTs and IIEF-5 values whereas there was a significant decrease in IPSS (p<0.01).

**Conclusion:**

A daily dose of 5mg tadalafil can be safely used in the treatment of erectile dysfunction and LUTS, that prolongs the ejaculatory latency time.

## INTRODUCTION

Premature ejaculation (PE) is considered one of the most common sexual function disorders in men with a prevalence of 9-30% (1-5). PE is defined as ejaculation with minimal sexual stimulation before or shortly after penetration, resulting in anxiety and distress. Patients have minimal or no voluntary control over PE (5). There are two types of PE: lifelong or primary, and acquired or secondary (5). Based on modern evidence, the causes of PE have been found to be psychogenic and performance anxiety (1, 5). Organic factors have been suggested as significant predictors of PE (2, 6). Genetic factors have also been listed among the factors affecting lifelong PE (7). Other common organic factors that have an impact on acquired PE include hormonal abnormalities (2), prostatitis (6), and erectile dysfunction (ED) (8). It has been reported that in many cases of lifelong PE, the men do not suffer from ED (9); however, approximately one third of the patients with ED have PE (10). Similarly, in a recent large-scale survey in the Asian-Pacific region administered to 4997 heterosexual men aged 18 to 65 years in a stable sexual relationship, ED was found to accompany PE in more than 30% of the respondents (11).

Many studies have suggested that assessing the effect of PE treatment is to measure the time taken to achieve ejaculation using the intra-vaginal ejaculation latency time (IELT). IELT is based on self-report and measured by a chronometer. It has 80% specificity and sensitivity for PE (12). Behavioral and pharmacological therapies are the common treatment options for PE. Behavioral therapy includes several techniques such as squeezing and start-stop methods but many couples have reported these to be inadequate. The first choice in pharmacological therapy is the use of serotonin reuptake inhibitors (SSRIs) (e.g., citalopram, sertraline, fluoxetine, dapoxetine or paroxetine); however, other options include phosphodiesterase type 5 (PDE 5) inhibitor therapy (tadalafil or sildenafil), topical desensitizing agents (prilocaine or lidocaine) and other agents (tramadol or pindolol) (13). PDE5 inhibitors are frequently used in the treatment of ED and clinical studies have reported their positive effect on patients with PE (14-16). In a recent study, a daily dose of 5mg tadalafil has been shown to significantly increase IELT in patients diagnosed with lifelong PE (17). However, to our knowledge, there is no study in the literature that determined the effect of tadalafil 5mg daily on ejaculatory time in patients with ED.

The current study investigated the effect of 5mg daily tadalafil treatment on the time taken to achieve ejaculation, erectile function and lower urinary tract symptoms in patients diagnosed with ED.

## MATERIALS AND METHODS

A total of 60 patients who were referred to the urology policlinic of the hospital with the complaint of erectile dysfunction between January 2015 and January 2016 were included in the study. The study was approved by the local ethics committee of Erzincan University and all patients gave informed consent for the treatment. All patients reported to be heterosexual and in a stable sexual relationship for more than six months. The exclusion criteria were neurological disorders such as depression, Parkinson’s disease, diabetic neuropathy, and cerebrovascular damage, an active urinary system infection, history of chronic prostate; alcohol, drug or substance abuse, organic diseases limiting the use of PDE5 inhibitors, pelvic trauma, anemia, thyroid disease, hypogonadism (total testosterone) end-stage renal failure; and having used medication affecting the sex hormone and/or vitamin metabolism or for the treatment of PE and ED within the last three months. The information related to patient’s age, duration of sexual dysfunction, smoking status, and sexual and medical history was obtained and a complete physical examination was performed on all patients. PE was assessed by IELT, which is defined as the time from vaginal intromission to intravaginal ejaculation (18). IELT was measured using a self-report method. It was measured by the female sexual partner using a stopwatch and expressed in minutes. If ejaculation occurred before or during penis vaginal intromission, it was defined as 0 minute. The same company calibrated all the stopwatches (12). The erectile functions of the patients were evaluated using the five-item international index of erectile function questionnaire (IIEF-5). According to their IIEF-5 scores, the ED patients were divided into three groups as severe ED (score: 1-7), moderate ED (8-11) and mild ED (11-21). The patient’s intra-vaginal ejaculation times were recorded. The lower urinary system symptoms (LUTS) of the patients were assessed using the international prostate symptom score (IPSS). Following fasting for 12 h, at 8 a.m., blood samples for the laboratory tests were obtained to measure the levels of fasting blood glucose (FBG), total testosterone (TT), triglycerides (TG), low-density lipoprotein cholesterol (LDL-C), and high-density lipoprotein cholesterol (HDL-C). The accepted normal values were: TT: 271-965ng dL^-1^, FBG: 70-110mg dL^-1^, TG: <150mg dL^-1^, LDL-C: <130mg dL^-1^ and HDL-C: >40mg dL^-1^.

For the treatment of ED, the patients were prescribed 5mg tadalafil daily for three months. At the end of this period, the patients were re-evaluated using IIEF-5, IELT and IPSS. In addition, the side effects of the treatment were recorded and the patient’s baseline and post-treatment scores were compared.

### Statistical analysis

A power analysis was conducted, in which the Biostatistics power of 80.193% was evaluated and the sample width was determined as a minimum of 19 individuals in each group. The statistical software SPSS (Statistical Package for Social Sciences, Version 20, Chicago IL, USA) was used for calculations. All values were presented as mean±standard deviation, means (maximum-minimum), percentages and frequencies. The results of the homogeneity (Levene’s Test) and normality tests (Shapiro Wilk) were used to decide which statistical methods had to be applied in the comparison of the study groups. Groups that were normally distributed and those with homogeneous variances were compared using the Student’s t test, and three or more groups were compared by the Analysis of Variance. According to the results of these tests, parametric test assumptions were not available for some of the variables and therefore the comparisons of two independent groups were performed by Mann-Whitney U test, and the comparisons of three independent groups were performed using Kruskal-Wallis test. For the multiple comparison tests, the adjusted Bonferroni method was used. The repeated measures of analysis of variance were analyzed by Mauchy’s sphericity test and Box’s Test of Equality of Covariance Matrices. For comparisons of the means of repeated measures, the Repeated Measures Analysis of Variance was used. When the parametric tests (factorial design for repeated measures analysis) did not meet the preconditions, methods by Greenhouse-Geisser (1959) or Huynh-Feldt (1976) were used for corrections to the Degrees of Freedom or Friedman Test. The Corrected Bonferroni test was used in multiple comparisons. The categorical data was analyzed with Fischer’s Exact Test and chi-square test. p values of <0.05 and <0.01 were considered statistically significant.

## RESULTS

The mean age of the 60 participants was 50.4±7.9 (range 36–67). The mean serum total testosterone, fasting blood sugar, total cholesterol, LDL-C, HDL-C were found to be 444.6±178.6ng/dL^-1^ (310–900), 104 (80-260) mg/dL^-1^,188.7±29.6mg/dL^-1^, 111.9± 32.4mg/dL^-1^, and 43.2±9mg/dL^-1^, respectively (Table-1). The mean baseline scores were 2.2±1.4 for IELTs, 9.5±3.7 for IIEF-5, and 14.1±4.5 for IPSS. At the end of the three-month tadalafil treatment, the patient’s scores were found to be 3.4±1.9, 16.1±4.7 and 10.4±3.8 for IELTs, IIEF-5 and IPSS, respectively (Table-2). The results indicated a statistical improvement in all parameters (p=<0.001). The pre- and post-treatment scores of the patients were compared according to the severe, moderate and mild ED groups. In all three groups, a statistically significant difference was found between the pre- and post-treatment values of IPSS variables (p<0.01) and a statistically significant difference was found between the pre- and post-treatment values of IELT variables (p<0.01) (Table-3). However, there was no significant difference between the ED groups in terms of the baseline and post-treatment values of IPSS (p=0.10; p=0.23) or IELT (p=0.83; p=0.48).

**Table 1:** Clinical data and fasting endocrine values of the participants.

Characteristic	Patients (n:60)
Age (year) ^*^	50.4±7.9
Total Testosterone (ng dL^-1^) ^*^	444.6±178.6
Total Cholesterol ( mg/dL^-1^) ^*^	188.7±29.6
Fasting blood sugar (mg dL^-1^) ^*^	104 (80-360)
HDL (mg dL^-1^) ^*^	43.2± 9
LDL (mg dL^-1^) ^*^	111.9± 32.4
Hypertension (%)	33.9
Smoking (%)	45.8
DM (%)	15.0

**LDL cholesterol** = Low-density lipoprotein cholesterol; **HDL cholesterol** = High-density lipoprotein cholesterol; **DM** = Diabetes mellitus.

* Mean±SD

**Table 2:** Baseline and post tadalafil 5 mg daily treatment IELT, IPSS and IIEF-5 scores of patients.

Variables	Pre-treatment	Post-treatment	p value*
IIEF-5	9.5±3.7	16.1±4.7	<0.001
IPSS	14.1±4.5	10.4±3.8	<0.001
IELT(min)	2.2±1.4	3.4±1.9	<0.001

**IIEF-5** = International Index of Erectile Function-5; **IPSS =** International prostate symptom score; **IELT** = intravaginal ejaculation latency time

*p values were derived from the statistical analysis using the independent t-test.

**Table 3:** Comparison of ED groups in terms of IPSS and IELT scores before and after tadalafil 5 mg daily treatment.

Group		IPSS_PRE	IPSS_POST	p	IELT_PRE (min)	IELT_POST (min)	p
severe ED	N	20	20	0.001**	20	20	0.001**
	Mean	15.70	11.20		2.30	3.10	
	Std. Deviation	3.83	2.97		1.17	1.41	
moderate ED	N	22	22	0.002**	22	22	0.001**
	Mean	13.95	10.64		2.09	3.50	
	Std. Deviation	5.35	4.74		1.48	2.32	
mild ED	N	18	18	0.001**	18	18	0.001**
	Mean	12.47	9.12		2.35	3.88	
	Std. Deviation	3.79	3.04		1.62	2.00	
Total	N	60	60	0.001**	60	60	0.001**
	Mean	14.12	10.39		2.24	3.47	
	Std. Deviation	4.56	3.78		1.41	1.95	
p		0,10	0.23		0.83	0.48	

**p<0.01

Table-2 presents the mean pre- and post-treatment IELT, IIEF-5, and IPSS of the patients. The common side effects were gastrointestinal problems or nausea in 6 patients (10%) and headache in 5 patients (8.3%). In addition, flushing was reported by 3 patients (5%) and muscle and lower back pain by 2 patients (3.3%). Most of the side effects disappeared over time.

## DISCUSSION

In this study, the effect of tadalafil 5mg daily treatment on ejaculation time, erectile function and lower urinary tract symptoms was investigated in patients diagnosed with ED. Corona et al. (19) recently conducted a meta-analysis on the relationship between PE and ED, and reported that PE increases the risk of ED approximately fourfold. In addition, this risk was found to be significantly higher in patients with depression and anxiety symptoms, followed by those with diabetes, hypertension and dyslipidemia. The IIEF scores of PE patients and the IELT scores of ED patients were found lower. According to the hypothesis proposed by Jannini et al. (8), PE and ED are part of a vicious cycle in which trying to control ejaculation reduces the instinctive level of stimulation resulting in ED. Similarly, in the effort to have an erection, the patient may try to increase his stimulation, which may result in PE. In order to test this hypothesis, Jannini et al. (8) retrospectively analyzed 184 cases (age range: 18-83), who were referred to the clinic with sexual function problems. The authors found that 29 cases with isolated ED had developed PE before ED. In the same study, 21 cases with isolated PE were found to be accompanied by, or have a history of mild to moderate ED (diagnosed using IIEF). Resulting in low satisfaction with sexual intercourse, PE can create psychological issues, which may lead to the development of ED.

PE can also develop secondarily to the increased stimulation for the creation and maintenance of erection in ED patients or accompanying anxiety (8). In parallel to this hypothesis, it was suggested that there is a higher risk of developing PE-associated ED for cases in which there is a direct correlation between ED and symptoms of anxiety or depression, and for those who do not have a stable sexual partner and experience stressful sexual relationships (19). Waldinger (9) suggested that ED is more commonly seen in patients with acquired PE compared to those with lifelong PE. Lifelong PE reduces sexual stimulation in patients, thus resulting in sexual intercourse accompanied by ED. On the other hand, McMohan et al. (20) used validated diagnostic tests and reported that 33% of the PE patients had been diagnosed with false positive ED. Today, the available PE treatment options include behavior therapy, topical anesthetics, and more predominantly SSRIs. However, studies concerning PDE5 inhibitors have also reported the clinical efficiency of these drugs in the treatment of PE. Studies investigating the therapeutic effects of PDE5 inhibitors alone and in combination with SSRIs have reported the benefits of these inhibitors for PE treatment (14-17). In a well-designed, randomized and double blind study, sildenafil was compared to a placebo (21). The authors reported that sildenafil increased the perception of ejaculatory control and overall sexual satisfaction, and reduced the time between the first and second ejaculation; however, it did not significantly increase IELT. Other studies (22, 23) have demonstrated that the combination of PDE5 inhibitors and SSRIs are more efficient in increasing IELT and overall sexual satisfaction compared to the individual use of these medications. These studies used sildenafil 50mg as the main PDE5 inhibitor.

In a randomized study, Salonia et al. (24) compared the efficacy of sildenafil, various SSRIs and the pause-squeeze technique, and reported that sildenafil increased IELT and sexual satisfaction and reduced anxiety. In addition, sildenafil, clomipramine, paroxetine and the pause-squeeze technique were found to increase IELT by 1 to 15 min, 4 min, 3 min, 4 min and 3 min, respectively in comparison to the baseline values (24). Recently, Ozcan et al. (17) reported a significant increase in IELT of patients with lifelong PE following 5mg daily tadalafil treatment. Although the study had limitations in terms of the small sample size (30 patients) and the short duration of treatment (1 month), it is significant in terms of being the first report on 5mg daily tadalafil treatment. In this study it was reported that IELT increased approximately 2.5 min while in our study increased 1.2 min. Our results showed that tadalafil 5mg daily treatment led to statistically significant improvement in all the measured parameters. Our results are supported by Ozcan et al. (17) who demonstrated that tadalafil 5mg alone could significantly prolong IELT. At the same time, in our study, there was no statistically significant difference between the ED groups in terms of IELT and IPSS following tadalafil 5mg daily treatment. Mattos et al. (16) study involving effect of tadalafil (20mg) alone and in combination with fluoxetine (90mg) found that the increase in IELT was better in patients who received combined treatment compared with placebo, fluoxetine, or tadalafil alone.

In the treatment of PE, regarding the effect of PDE5 inhibitors, there are several mechanisms involved. All central and peripheral mechanisms are probably important but the particular role that each plays in delaying ejaculation is not known. However, the mechanism that is most speculated to be involved is the reduced sympathetic tone and smooth muscle dilatation. Aversa et al. (25) reported that PDE5 inhibitors display activities through central and peripheral mechanisms. The NO/cGMP signaling pathway is considered to control sexual behavior through a central effect. The possible mechanism of the PDE5 inhibitor action lessens the contracting response of vas deferens (VD), seminal vesicles (SV) and prostate and urethra. This creates a state of peripheral analgesia, which prolongs the duration of the erection and reduces the central sympathetic output (26). The results of these studies demonstrate that PDE5 inhibitors relax VD, SV and smooth muscle tissue in the prostate, and increase the duration of the erection and sexual confidence, resulting in increased overall sexual satisfaction.

Studies have suggested that LUTS cause erectile dysfunction (27) and ejaculatory problems (28). The pathophysiological links between LUTS and ED are not fully understood, and these conditions are suitable to therapy with phosphodiesterase type 5 inhibitors (PDE5-Is). Some studies have determined the role of phosphodiesterase type 5 inhibitors in the treatment of men with LUTS associated with benign prostatic enlargement. Yan et al. (29) conducted a meta-analysis on the use of PDE5 inhibitors in the treatment of LUTS and reported that these inhibitors reduced IPSS by 4.21 points. Similarly, in this study, we found a significant decrease of 4.3 points in IPSS and an increase in the IIEF-5 score after using tadalafil 5mg daily treatment. Oelke et al. study, a post-hoc analysis of four randomized studies in 1477 men, showed that patients treated with tadalafil 5mg once daily versus placebo presented a clinically-meaningful symptom improvement (decrease more than 3 points of total IPSS) (30). Wein et al. (28) study reported that LUTS caused ejaculatory problems. Alpha blockers drugs are very important for the treatment of LUTS. On the other hand, Akın et al. (31) showed that all this alpha-blocker drugs were statistically effective in preventing PE. The authors found that the IPSS score significantly decreased in all groups while there was a statistically significant increase in IELT and a decrease in premature ejaculation profile scores in all alpha-blocker drugs groups in the post-treatment period. In this study, it was observed that tadalafil 5mg daily treatment led to statistically significant improvement in IPSS and IELT. Similarly, Choi et al. (32) reported a significant change using in the LUTS+PE patients after tamsulosin administration according to the results of the premature ejaculation diagnostic tool (PEDT). The positive effect of tamsulosin on PE can be attributed to the decreased contractility of the seminal vesicle or the vas deferens by the drug itself. Furthermore, the improvement of PE could be secondarily affected by the improvement of LUTS. This effect was also demonstrated by Aversa et al. (25), who used PDE5 inhibitors to relax VD, SV and smooth muscle tissues in the prostate.

PE has been found to be related to comorbid disorders such as diabetes. El-Sakka et al. (33) showed that men with diabetes had a high prevalence of PE. Many patients with ED develop PE probably due to the need for intense stimulation or anxiety to initiate and maintain an erection (34). In our study, only 9 patients (15%) had DM and their fasting blood glucose was controlled.

The limitations of our study include a small sample size and the absence of a non-ED control or placebo group. Treatment with PDE5 inhibitors is significant not only in terms of the relationship between ED and PE but also due to improving the erection and reducing the ejaculation problems caused by LUTS. This study is a valuable contribution to the literature in terms of being the first study investigating the effect of tadalafil 5mg daily treatment on ejaculatory time in patients with ED.

Daily 5mg tadalafil treatment is considered to have beneficial effects on ED and PE patients. Therefore, we recommend the use of 5mg tadalafil once daily, specially in those men with PE with erectile dysfunction. Further studies must be conducted with a placebo-controlled larger series and a longer follow-up to contribute to the literature in terms of the effects of daily 5mg tadalafil treatment.
